# Assessment of the Impact of Wear of the Working Surface of Rolls on the Reduction of Energy and Environmental Demand for the Production of Flat Products: Methodological Approach

**DOI:** 10.3390/ma15062334

**Published:** 2022-03-21

**Authors:** Mariusz Niekurzak, Ewa Kubińska-Jabcoń

**Affiliations:** Faculty of Management, AGH University of Science and Technology, 30-067 Krakow, Poland; ejabcon@zarz.agh.edu.pl

**Keywords:** production planning, hot-rolling mill, waste of metallurgical rolls, steel, strip

## Abstract

An important element in the correct operation of the rolling mill is appropriate planning of the condition of the rolls because this factor constitutes a limiting element in the production process. In this work, with the aim of indicating the method of proper use of production tools–metallurgical rollers during their operation in a Polish rolling mill, the wear and tear of particular kinds of rollers built in the whole rolling set was determined. For this purpose, data were collected at the strip mill from grinding processes, production reports and roll files, while our statistical analysis, laboratory calculations and measurements were used. These data were used to perform computer calculations on the service life of metallurgical rollers installed in the rolling line. Wear mechanisms were identified in industrial practice. The characteristic features of roller wear were investigated using non-destructive tests, including eddy currents. The laboratory tests reproduced the wear mechanisms in very hot rolling rolls. The statistical method for determining the service life of working rolls indicated that their reconstruction is determined both by natural physical phenomena and inappropriate use in about 30% of cases, mainly in the F5 and F6 cages of the finishing unit. Calculations indicated the possibility of replacing the working rolls made of high chromium cast iron Hi-Cr with those made of HSS in the F5 and F6 cages, which will contribute to an increase in the durability of the rolls, a reduction in production costs and a decrease in the number of roll rebuildings. The service life of HSS rolls is 14,000–20,000 Mg of rolled material per 1 mm of wear on its surface in the radial direction, compared to 2000 Mg for rolls made of high chromium cast iron Hi-Cr. The constructed model may be a source of information for further analyses and decision-making processes supporting the management of metallurgical enterprises. On the basis of the constructed model, it was shown that the analyzed projects, depending on their type and technical specification, will bring measurable economic benefits in the form of reduced annual energy consumption and environmental benefits in the form of reduced carbon dioxide emissions into the atmosphere. The constructed model of the roll consumption, verified in the real conditions of the rolling mills, will contribute to the fulfillment of energy and emission obligations with the EU.

## 1. Introduction

In the process of steel strip production, the tool most responsible for steel strip quality are metallurgical rolls. However, metallurgical rolls are one of the most expensive tools used in the plastic processing of metals. Used in cold or hot rolling mills, they can produce flat or long products. Their quality depends on the microstructure of the material from which they are made, and on the calibration [1]. The microstructure depends primarily on the chemical composition of the material from which they are made and the heat treatment. With regard to the required quality of rolled strip, the condition of the rollers of the finishing unit is of paramount importance, as they have the greatest influence on the dimensional accuracy and quality of the strip [2]. During the production of steel strip, a significant problem is the high degree of wear of the rolls, especially working rolls, and thus there is a need for frequent rebuilding. The working rolls of the entire finishing unit are changed every 8–10 h and the thrust rolls every 2–3 weeks, depending on the quantity and assortment of rolled strip [3]. The continuous improvement of this tool (e.g., through the correct calibration of rolls) results in increasingly higher productivity and ensures the production of strips with a higher surface quality and more accurate geometric dimensions [4]. Failure to calibrate rolls correctly will result in even the best roll material being unable to handle excessive localized pressures or stress concentrations, requiring rebuilding or cracking after a short period of operation. New roll materials are continually being sought to improve the service life of rolls. Selecting the right chemical composition and heat treatment of the roll material affects its service life. Continuous improvement of this tool results in increasingly higher productivity and ensures the production of strips with higher surface quality and more accurate geometric dimensions [5].

In the literature, there are many works [6,7,8,9,10,11,12,13] on the potential of using RES technologies in the global energy mix, while apart from [7], there is little research on reducing CO_2_ emissions into the atmosphere by plants with a diversified production method, i.e., metallurgy. In Poland, the steel industry is responsible for 14% of total CO_2_ emissions. It is a branch of the economy that is still in an intensive production phase and due to the high energy demand for the production of steel, conventional energy (i.e., natural gas and electricity) cannot be replaced with energy from renewable sources. Therefore, solutions should be sought to optimize existing technologies that will help reduce CO_2_ emissions, and thus reduce emission allowances payments to production plants. These issues are described in detail in the paper: “Modeling of Energy Consumption and Reduction of Pollutant Emissions in a Walking Beam Furnace Using the Expert Method—Case Study” [7].

With all aspects in mind, the aim of this research was as follows: “The purpose of the work was to determine the amount of wear of the working surface of a barrel of rollers. Depending on the rolling program being implemented, it will enable the proper selection of the type of material for the implementation of the roll for installation in individual stands of the rolling unit, which will increase their durability and operating efficiency.” Achieving the goal of the work requires completion of the following tasks:

- Statistical analysis of the production results concerning the determination of the durability of rolls in the hot rolling mill for steel strips;
- Determination of the amount of roller wear in individual stands of the rolling unit;
- Determination of the wear indicators of rollers installed in the rolling unit, made of various types of materials;
- Performance of metallurgical tests and calculations in order to find a better material for rolls than is currently used;
- Determination of projected roll reconstruction times.

As metallurgical rolls are used in the global metallurgical industry, and there is no comprehensive work on determining the durability of rolls during the operational period of long-term production planning, an attempt was made to analyze this process in a broader manner using measuring techniques from the “Roll Grinding Management System”.

This work is partly cognitive and theoretical in nature, but it mostly has a practical dimension. It is a systematic presentation of the authors’ research and theoretical considerations to date, extended by some new detailed theoretical solutions and research performed in industrial conditions. Such considerations are necessary for a comprehensive process analysis, with the available specialist literature not providing sufficient information on this subject.

## 2. Review of the Literature on the Issue under Analysis

The analysis of roll wear in the various stages of the steel strip production process is a very complex issue. In order to determine the exact degree of wear and to locate those areas on its surface that are most exposed to damage, a comprehensive analysis of the steel strip production process is necessary. Computer calculations are increasingly being used in the analysis, allowing an approximate assessment of the degree of wear of working tools and enabling the determination of the location of areas on the surface of the rolls most cornered on their wear [14,15,16,17]. The analysis of these quantities allows for continuous research and improvement in the direction of extending the service life of rolls.

A major factor in increasing production costs in the hot strip mill is the frequent rebuilding of rolls. This problem is due to the insufficient service life of the working barrels of the rolls. These problems result in the rolling mill incurring huge consumption costs, mainly electric energy and natural gas [18]. These losses arise due to compulsory stoppages of the rolling mill to carry out the rebuilding of the rolls. The problem of determining the durability of rolls still constitutes the main objective of metallurgical research. The necessity of using increasingly better quality rolls suitable for work in difficult exploitation conditions is a challenge for contemporary science. Due to the importance of reducing production costs of the finished product (i.e., steel strip) and maintaining its dimensions within narrow tolerance limits, an increasing number of researchers undertake each year to publish their work. Every year, many meetings, seminars and scientific conferences devoted to this subject are organized, with increasing documentation on the web. Many authors [19,20,21,22,23,24,25,26,27] have focused their research on the search for materials for metallurgical rolls with high resistance to wear specific to their production.

However, specialized literature does not provide sufficient information on the verification of a specific grade of roller in industrial conditions during the period of realization of short- and long-term production. Extending the life of rolls contributes to a significant increase in rolling times, increasing production efficiency and reducing mill costs associated with the need for frequent replacement and remanufacturing of rolls. In the case of other steel companies, if work has been undertaken in these areas, it was not reflected in scientific publications.

The basic conclusion drawn from a literature review of the past three decades is that there is insufficient literature documenting the achievements of experimental research on the subject matter covered in this manuscript. Since metallurgical rollers are used in the global metallurgical industry, and there is a lack of comprehensive work on determining the durability of rolls during the service life of long-term production planning, an attempt has been made to analyze this process more extensively.

### Factors Influencing Roller Wear

Rolls are the basic tool of rolling mills, as they directly form a strip of metal, giving it successive shapes, until the finished product is of sufficiently precise dimensions and has a satisfactory surface. The wear of rolls is one of the most important issues of the economics of the rolling process and is recognized as one of the basic material criteria, with the amount of rolled material making it necessary to replace the rolls [28]. Roller wear is influenced by the rolling temperature, the size of the dies used (which depend on the rolling program), the chemical composition of the rolls and the natural physical phenomena occurring during the rolling process.

To be considered of good quality, rolled products must have dimensions falling within strictly defined tolerances given in appropriate standards or agreements and meet the requirements for surface quality and technological properties. These factors are decisive in determining the point at which rolls lose their ability to produce a product that is correct in terms of accuracy and surface quality. Thus, they directly determine the permissible amount of wear of the roll barrel surface. As the quantity of rolled steel strip increases, the condition of the roll barrel surface deteriorates, which first manifests itself in the loss of the required surface smoothness, and thus rolled products, and then in the variation of the band thickness on its cross-section. The wear of the working surface of the roll barrel during rolling results from the physical phenomena of the rolling process, which are made visible by the action of three fundamental mechanisms [29,30,31,32,33]:

1. Abrasion of the barrel surface as a result of sliding with a deformed hot strip of metal, moving in relation to its surface;
2. Mechanical fatigue of the roll surface layer due to cyclic changes in mechanical stress induced by the rolled material;
3. Thermal fatigue of the surface layer caused by cyclic local temperature changes resulting in stresses between areas of different temperatures.

During rolling, periodically varying loads act on the rolls. At high speeds, the action of forces can be treated as dynamic impacts on an inert mass. At excessive stresses that exceed the strength limit of the material, the roll breaks at the point of force action. Damage to the roll barrel surface is the most common cause affecting the reduction of roll life. The tribology of the roll surface is also affected by increased slip and unit pressures of metal on the roll, as well as decreasing the accuracy of cleaning the band from the scale. As a result of these factors, the amount of rolled metal, which causes wear of the rolls, changes during the process from the first to the last frame. Due to the uneven wear and tear of the rolls and the deterioration of their surface condition, the rolls must be periodically replaced and subjected to grinding in order to obtain the appropriate shape and remove the effects of wear and tear [34,35,36,37].

When explaining the mechanisms of wear and tear of rolls, in order to determine the influence of individual mechanisms, it is necessary to consider the effect of factors always referring to the action of a certain, equal quantity of material.

The abrasion of the barrel surface is caused by friction between the working surfaces of the cylinder and the rolled material. This mechanism depends on the smoothness of the surface, shape, crumple as a factor determining the pressure value, the amount of lead, lag, temperature and speed. The increase in wear of the rolls by abrasion causes the presence of scale by increasing the coefficient of friction. Moreover, the scale causes the surface defects on the rolled material. Abrasion is a major cause of wear on rolls in the end stands. There are ways to reduce the abrasion caused by hard iron oxides. They are:

- Maintaining the surface temperature of the rolled metal below 900 °C, a condition that often cannot be met due to the need to obtain the required final rolling temperature;
- The use of nozzle spraying to lower the surface temperature of the rolled strip;
- Reducing the rate of scale formation by creating a protective atmosphere around the surface of the rolled material;
- The formation of hard alloys on the surface of the rolls.

This state is influenced by changing normal loads, tangential loads and internal stresses. The size of the normal and tangential forces determines the abrasion of the barrel surface and the formation of chipping when the stress value exceeds the fatigue strength of the cylinder metal.

Thermal fatigue is caused by cyclic changes in the temperature of the surface layer of the cylinder. Temperature changes caused by the intense heating and cooling of the roller surface create stresses, which can lead to the formation of thermal cracks. The temperature of the surface layer of the cylinder reaches 500–700 °C, which causes high thermal stresses, lowering the hardness and strength of this layer of the cylinder, and consequently lowering the resistance wear roller. Destruction of the cylinder surface due to thermal fatigue results in the constant formation and growth of subsurface cracks, causing the material to fall off in the following ways: chipping, peeling and fractures from the surface layer of the cylinder. The impact of cracks is magnified by other factors, such as mechanical stresses and the effect of cooling water, which turns into steam when it comes into contact with the heated metal, and the resulting high pressure increases the existing cracks. During the rolling process, parameters such as temperature, pressure and load duration change. The result is that the amount of metal rolled, which causes the wear of the rolls, changes as the process proceeds from the first stand to the last stand.

Summarizing the considerations made so far, it can be said that the surfaces of the work rolls of hot rolling mills wear out as a result of contact with the heated rolled strip and with the support rolls. Consumption is affected by the following factors:

- Roll material and chemical composition of rolled steel;
- Total rolled length during one installation;
- Temperature of the rolled material and rolls;
- Value of the unit and total metal pressure on the rollers,
- Rolling speed;
- Value of the slip of the rolled metal in relation to the rolls in the rolling gap;
- Surface quality of the rolled material;
- Surface condition and hardness of rolls.

The greater the hardness of the rolls, the less their wear. Work rollers wear almost over the entire surface of the barrel, but most intensively in the center of the barrel. Due to the uneven wear of the rolls and the deterioration of their surface condition, the rolls should be periodically replaced and grinded in order to give the correct shape and remove the effects of wear.

The roll is considered technically sound if the measured results of its diameter and hardness in specially designated places on the roll surface are within a specified tolerance range and meet the requirements in terms of accepted standards.

## 3. Materials and Methods

### 3.1. Statistical Analysis of Wear and Tear of Rolls

Strength properties of rolls in terms of wear resistance during rolling are subject to certain changes. Against this background, it is necessary to check and possibly correct the size of the rolled batch of material. This mainly happens when the material grade of the rolls is changed. The quantity of rolled material in a given rolling mill can be corrected on the basis of statistical research. The statistical surveys are based on existing data in the rolling mill, recorded in files and reports on the operation of the rolls. These tests do not require knowledge of specific changes in roll quality and calibration, and can be performed at any time. Based on theoretical premises, it was assumed that the natural wear of rolls is subject to the laws of the normal distribution. This assumption is based on the claim that the error distribution tends to be normal when the error is caused by the sum of similarly influencing mutually independent factors. For example, during hot rolling, factors include strand temperature, roll orientation, calibration scheme, rolling speed, type of rolled material, roll hardness, etc. It was assumed in the considerations that the roll life was determined by the Mg amount of the steel rolled between two consecutive roll turns. The natural wear of rolls is understood to mean a process in which rolls are only considered worn as a result of the loss of their ability to give the strands the correct surface quality and the required shape. In practical conditions, the rolling process may be interrupted by organizational factors resulting, inter alia, from roll rebuild schedules. As a result, in practice the spread of masses followed by conversion is narrowed.

Based on the results of the tests of wear and tear of rolls, the service life of rolls installed in the whole rolling assembly of the analyzed rolling mill was determined by statistical calculations. According to statistical principles, it was assumed that the natural wear and tear of rolls is subject to the laws of the normal distribution. Taking this into account, two hypotheses were assumed:

**Hypothesis** **0** **(H0).** *If the examined distribution is normal, then the physical phenomena prevailing in the rolling process at the contact between the rolled metal and the rolls determine the replacement of the rolls.*

**Hypothesis** **1** **(H1).** *In addition, the alternative hypothesis assumes that if the studied distribution is not normal, then the rolls are not used properly.*

### 3.2. Quantitative Analysis of Roller Wear

By analyzing the data collected in roll cards, production reports, in-service roll turnover reports from individual months and other documents, the roll-wear were determined. The purpose of this research was to determine the influence of the length of the rolling campaign on the actual wear of individual roll types.

The scope of the developed data concerned the wear of rollers in individual cages of the finishing unit, designated as F1–F6 in the years 2020–2021. During the analyzed period, rollers made of the following steel were operated: high-speed steel—HSS (High Speed Steel), high chromium cast iron—Hi-Cr (High Chromium Cast Iron) and modified alloy cast iron of the EICDP and ICDP (Enhanced Chromium Steel) types. Rollers made of the aforementioned materials ran 53 rolling campaigns in total and rolled 1500 Gg in 2020 and 1100 Gg in 2021 of general purpose unalloyed and alloyed steel strip [38].

The two-year period for which data was analyzed was not homogeneous in terms of production. During this period, there were times when low-alloy steels were rolled and times when they were not rolled, but the rolling conditions used did not change significantly. As a measure of the degree of utilization of the roll, the *Q* index used in metallurgy was adopted, determined from the following formula [7,38]:

*Q* = *Z*/*U* (1)

where *Z* is the amount of rolled steel, Mg, and *U* is the reduction in roll weight due to rolling and calibrations, kg.

The total wear and tear of rolls, referred to as natural wear and tear, is the sum of metal wear and tear of the barrel of rolls expressed in kg as a result of compulsory rolling during the whole period of operation of the roll, i.e., from the first deposit to withdrawal for scrap. Collecting and analyzing data on the wear and tear of rolls provides each rolling mill with very valuable information, making it possible to determine the times for rebuilding the rolls. This considerably improves the production planning process of the whole technological line and contributes to the reduction of operating costs.

Analyzing the annual reports, it clearly shows that the rolls wear in proportion to the amount of rolled material. Roller wear depends on the production program. The wider and thicker that products are rolled, the lower the wear of the rolls, and the narrower and thinner products are rolled (the width b:L cylinder barrel length ratio is small), the greater the wear of the rolls. In order to ensure conditions for the uniform wear of the rolls, and thus to obtain the correct profile of the rolled strips, the schedule of wide and narrow strips should be properly selected and the sequence of rolling thick and thin strips should be planned. To ensure rational operation of rolls and proper product deviations, strip mills should follow the principle of consecutive rolling cycles and arranging rolling campaigns. First, the widest strands (first cycle) are rolled, followed by the intermediate strands (second cycle) and finally the narrowest strands (third cycle). By following these rules, the number of roll changeovers can be reduced five times during the implementation of production plans.

### 3.3. Hardness Measurements of HSS Sample

The tests were performed on a sample cut from a laboratory ingot of HSS with symbol HSS, mass 40 kg and chemical composition given in Table 1.

Hardness tests were performed on samples cut from the upper and lower parts of the cylinder. Hardness measurements were made using the Rockwell method. Metallographic tests were performed for samples taken from areas of clearly different hardness. Metallographic observations and photos were made on the metallographic microscope. Technological considerations (machining) allowed the taking of samples (in the shape of rings) from the top and the lower part of the cylinder, but only to a depth of 75 mm. In most cases, this depth comprised both the hardened layer and the roll core. 

### 3.4. The Principle of Using the Effect of Eddy Currents in Non-Destructive Testing of Materials

**Figure 1:**
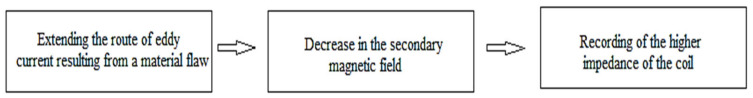
The sequence of phenomena while detecting inconsistency in a material. Source: own, based on [38].

In order to achieve a reduction in rolling mill production costs, a reduction in the number of roll rebuilds required and optimum utilization of their working area, calculations were made using the “Roll Grinding Management System”. The purpose of the calculations was to determine, using eddy current testing, the type of defects and their location in the material structure of rolls made of Hi-Cr cast iron. The “Roll Grinding Management System” program is connected to a roll grinding machine with, roughing and finishing of the rolls to give them the desired profile within a narrow tolerance range. It also allows us to determine their tribological properties using the eddy current method. In addition, the aim of the calculations was to determine the effects of replacing the work rolls made of high chromium cast iron Hi-Cr, currently operating in cages F5 and F6 of the finishing unit, with those made of a new generation of high-speed steel HSS, characterized by superior mechanical and technological properties. The detection of discontinuities in the material follows the sequence of phenomena shown in Figure 1.

The method that uses the effect of eddy currents to examine the state of a material in a non-destructive manner involves detecting, using an alternating magnetic field, local differences in physical properties of the material of the tested elements that cause a change in the intensity of these currents. In practice, this is performed in such a way that the tested element, with specific dimensions, made of a material with a given electrical conductivity and magnetic permeability, is subjected to the action of an alternating electromagnetic field in the tested material and receives the material’s reaction through a test probe and eddy current flaw detector. The analysis of the value of changes in the electromagnetic field, amplitude and phase shift of voltage and intensity allows for a very precise assessment of the condition of the tested material, any discontinuities such as cracks, erosive or corrosive defects, and the assessment of their size and depth.

If the coil is moved at a constant speed at a constant distance from the surface of the object, all changes in the cohesion or thickness of the material being tested affect the current flow and consequently the magnetic field, magnitude and phase of the voltage in the coil. This method makes it possible to detect the most dangerous discontinuities extending to or lying close to the surface, as well as discontinuities under the coating layer and those located in individual layers of multilayer objects. Cracks with depths from about 0.1 mm, widths from about 0.0005 mm and lengths from about 0.4 mm are detected. In this way, it is possible to detect and locate a material defect present in a given roll.

If you measure the height of the top and bottom vertices on the surface of the roll with respect to the zero position, you obtain key information about the technical condition of the roll under test, defined by its non-roundness *N* (*α*) [7,38]:

(2) $$N\left(\alpha \right)=\frac{E\left(\alpha \right)+Q\left(\alpha \right)}{2}$$

where *N* (*α*) is the roundness of the roll, *E* (*α*) is the height of the top peak of the roll and *Q* (*α*) is the height of the bottom peak of the roll.

## 4. Results and Discussion

### 4.1. Statistical Analysis of Roll Wear

As a result of the statistical analysis performed (Table 2) using the test of concordance at a significance level of *α* = 0.05, it was found that of 100 cases involving the process of rolling strips of different grades of rolled steel, intended for various industries, 10% of the cases did not confirm the assumption of normality of the distribution. These cases, according to the assumption, were used to determine the degree of proper underutilization of the roll.

$\chi_{\alpha }^{2}$—distribution of a random variable.
$\chi_{obl}^{2}$—computed significance level assessment.
$\chi_{\alpha }^{2}>\chi_{obl}^{2}$—no reason to reject the null hypothesis.
$\chi_{\alpha }^{2}\leq \chi_{obl}^{2}$—the null hypothesis should be rejected.
α = 0.05—materiality level.

The proposed research method makes it possible to determine the durability of rolls in individual cages and, at the same time, gives the possibility of correcting the size of the rolled material batches. It enables the development of more accurate assumptions for the rational management of rolls, allows for a better selection of appropriate materials for rolls and, moreover, makes it possible to develop an appropriate schedule of rolls reconstruction.

### 4.2. Quantitative Analysis of Roll Wear

Based on automated grinding processes whose grinders record actual measurements to the nearest 0.0001 mm, the roll-wear levels for 2020 and 2021 are summarized in Table 3.

From analyzing the indices determined during the tests, it can be observed that the greatest wear is exhibited by rollers made of high chromium cast iron Hi-Cr built in the F5 and F6 cages of the finishing unit. Their wear in both 2020 and 2021 exceeded the permissible limit tolerances established in the standards given by the manufacturer of this type of roller. Consequently, this led to a reduction in the efficiency of the rolling process and an increase in production costs. Higher consumption depended on the implemented production program. Narrower and thinner strips were rolled in 2020 and 2021, resulting in higher roller wear. Compared to previous years, where wider and thicker strips were rolled, the durability of the rolls was greater. In summary, higher consumption in the analyzed case does not depend on the production volume but on the implemented production program.

### 4.3. Hardness Measurements of HSS Sample

Rolls made from this HSS are increasingly replacing cast iron rolls with an indeterminate hardening layer and are a continuation of the development of cast iron high chromium rolls. The change in their structure consists of replacing part of the chromium with other carbide-forming elements such as tungsten, titanium, niobium and vanadium, with the aim of improving the mechanical and technological properties of the steel [39,40,41]. The basis for the heat treatment of the rolls was the tempering procedure. Industrial tests have shown the suitability of the performed treatment immediately after hardening. This treatment made it possible to achieve the most favorable hardness distribution on the cylinder cross-section and the required hardness on the barrel surface, which was mainly expressed by their 1.5–2 times longer life. This heat treatment made it possible to harden a thicker layer of the roller, which allows for less wear of the rollers and a longer service life in one structure. The types of heat treatment performed and the resulting hardnesses are given in Table 4.

It can be seen from Table 4 that after casting and cooling to an ambient temperature, the tested high-speed steel shows a very high hardness: 60 HRC (84 HSh). Ingots of the test steel were further softened by heating to 700 °C, held at this temperature for 8 h, and then slowly cooled with an oven. The test ingot specimens were then hardened and tempered twice at 580–600 °C for 3 h with subsequent cooling in air. This resulted in a hardness of 71–75 HSh.

The volume proportion and morphological characteristics of the carbide phase have a decisive influence on the quality of the rolls. The optimal solution for the purposes of the analyzed studies turned out to be a reticulated arrangement of carbides obtained as a result of the application of incomplete normalizing annealing. Self-healing of the surface layer of rolls with such microstructure, due to easy removal of the so-called white layer from the surface of the roll [33], practically prevents the formation of a network of micro-cracks and fractures, with its hardness measured by the amount of Mg of the rolled product depending on the stereological parameters of the carbide phase and the properties of the matrix. If the chemical composition of the roll material, with carbides in the form of a lattice optimal from the tribological point of view, makes it possible to change the hardness of its matrix, it becomes possible to produce good, efficient rolls for both cold and hot rolling mills of steel sheets and strips.

### 4.4. The Principle of Using the Effect of Eddy Currents in Non-Destructive Testing of Materials

**Figure 2:**
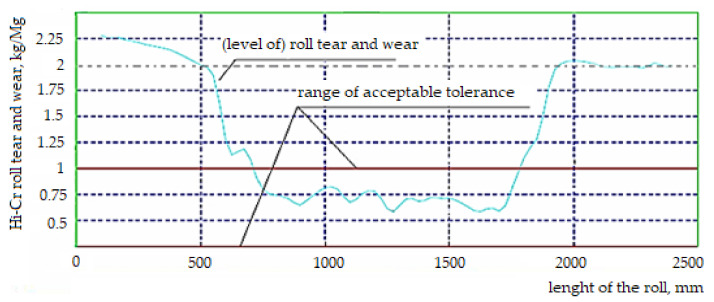
Diagram of high chromium cast rolls local tear and wear levels. Source: own, based on [38].

After entering all the roll parameters into the computer system and comparing them with the range of actual tolerances, the tested curve of the roll operation in the rolling process moves outside the range of the established permissible tolerance determined for its optimal operation, as shown in Figure 2.

During the tests performed, the cast iron roller, after one rolling campaign (2000 Mg), showed worse surface quality parameters. This was because rollers of this type were installed in the first stands of the rolling unit in which the most difficult working conditions occur, e.g., due to the significant reduction of the strand cross-section. The defects appearing on the working surface of the cylinder can be determined based on the fault map suggested by the program using the error charts generated from the theoretical model in comparison with the obtained profile. To perform a more detailed analysis, the surface of the roller is conventionally divided into sectors along its radius and stations along its length (Table 5). The level of specified roller wear is indicated for each separate sector cell and station of the specified roller. Based on the defect maps, it is possible to determine the types of defects in the roll, such as cracks and run-outs, which are caused by frictional phenomena and affect the wear of the roll.

**Figure 3:**
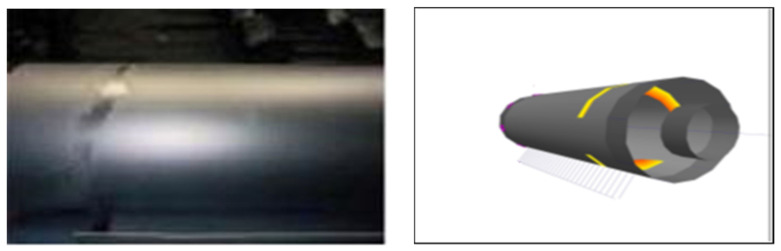
Roller indentation defect mapped in 3D. Source: own, based on [38].

The darker boxes with bold numbers in Table 5 indicate the locations of the resulting material defects. Figure 3 shows the fracture defect of a Hi-Cr type roller that was operated for a certain period in the F5 and F6 cages of the finishing unit. Using the calculations program, the location (i.e., sector and station of its defect) were located.

**Figure 4:**
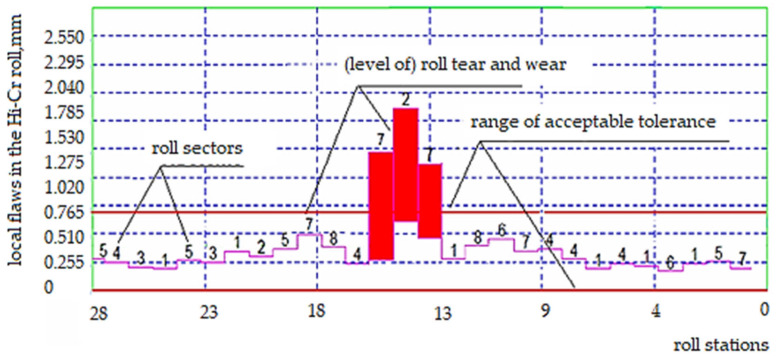
Diagram of faults level generated based on the map of faults for the high chromium Hi-Cr cast roll. Source: own, based on [38].

From the diagram in Figure 4, it can be assumed that the greatest wear, which occurred in the central part of the cylinder, proves that this type of cylinder is not wearing evenly over its entire surface. This may be due to the impact of the rolling program in which thinner and longer products were rolled.

**Figure 5:**
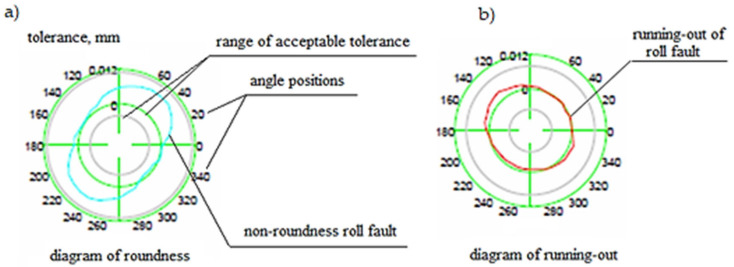
Diagram of defects of rolls made of high chromium cast iron Hi-Cr: (**a**) irregularities, (**b**) run-out. Source: own, based on [38].

On the other hand, the polar diagram shown in Figure 5 provides the necessary information related to latent defects occurring under the working surface of the barrel of the cylinder and defects in the form of out-of-roundness shown for different angular positions.

**Figure 6:**
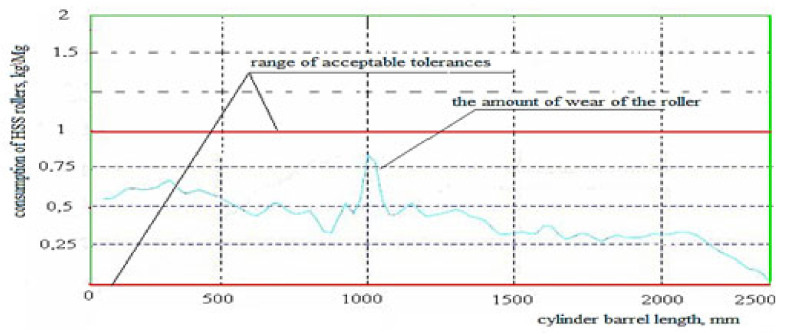
Graph volume of local consumption for HSS rolling type. Source: own, based on [38].

If the sum of the height of the vertices calculated from Formula (2) produces a circle, this means that the roll is perfectly round and does not cause run-out, which leads to accelerated tribological wear. On the other hand, if the calculated sum of the height of the vertices gives an ellipse, this means that the roller is not round and has a run-out defect, which results in the propagation of cracks leading to premature wear. By analyzing the graph of the modified Hi-Cr alloyed cast iron roll shown in Figure 5 and using the principle of superposition of effects, which considers both the roundness errors caused by the roll’s journals and tusks, it was determined that an elliptical shape was being dealt with here. Therefore, the roll has pronounced run-outs beyond the allowable range of the established tolerance, which is noticeable mainly in frames F5 and F6 of the finishing assembly. These rolls often have to be rebuilt, including replacing them with new ones, which increases production costs and disorganizes the entire work planning system of the rolling mill department. By using analogous reasoning for the HSS roll, it can be seen that a roll made from HSS has greater strength and wear resistance under the same operating conditions as a Hi-Cr roll [42,43]. The graph in Figure 6 clearly demonstrates that the dimensions of the HSS rolling campaign roll of the same length as that made of Hi-Cr cast iron are further within the acceptable tolerances.

This means that it can operate on a large scale, with the ability to roll an additional 20,000 Mg of strip without having to rebuild it. In order to extend the operational life of the rolls and to reduce their production costs, HSS rolls can be used, especially in F5 and F6 cages, which is where they are most vulnerable to wear. On the basis of the map of defects, presented in Table 6 and obtained during the conducted calculations, it can be concluded that only small local defects marked with a grey color and a bold number, which appeared in the same working conditions as the case of rolls made of high chromium steel, are noticeable in station 5.

**Figure 7:**
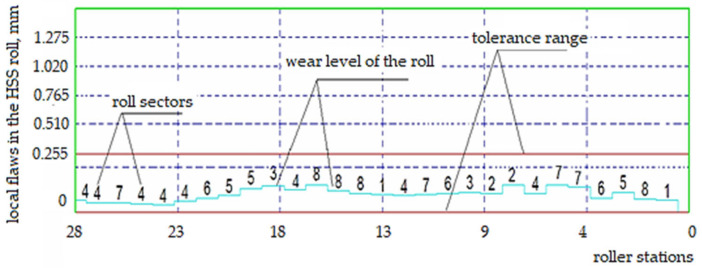
Graph-level defects generated on the basis of faults map of type HSS. Source: own, based on [38].

These disadvantages, obtained after analyzing the annual reports, were due to the high temperatures occurring during the rolling process. However, these disadvantages do not require rebuilding the rollers, as is shown in Figure 7.

**Figure 8:**
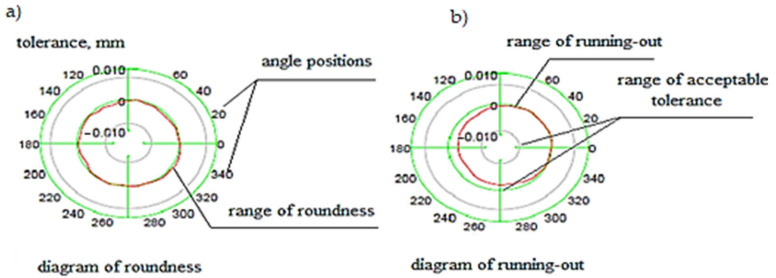
Diagram of defects of rolls made of HSS high-speed steel: (**a**) out of roundness, (**b**) run-out. Source: own, based on [38].

Figure 7 clearly shows that the defect in sector 5 of the roll is within acceptable tolerances. This does not disturb the mill operation as the working surface of the roll barrel wears uniformly along its length. The polar diagrams for the HSS roll shown in Figure 8 show that it is circular in shape and therefore does not have surface defects or hidden run-out defects that cause roll cracking.

**Figure 9:**
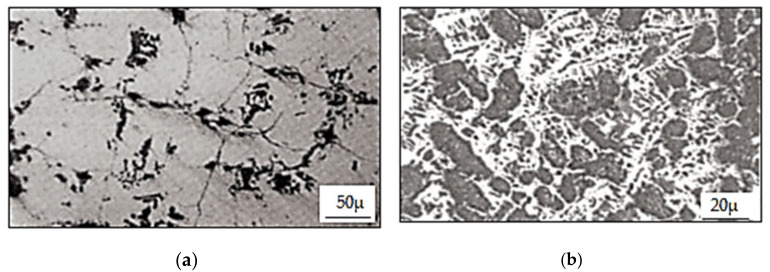
Cross-section through a hardened cast iron cylinder after a campaign: (**a**) without etching, (**b**) with etching (2% Nital) [38].

**Figure 10:**
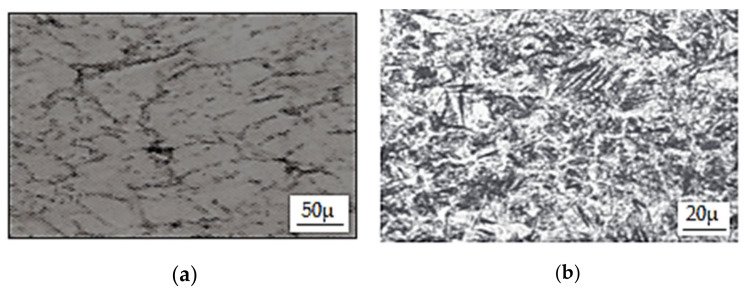
Cross-section through a high-speed steel cylinder after a campaign: (**a**) without etching, (**b**) with etching (2% Nital) [38].

The values of defects determined on the basis of the method of superposition of effects allow us to conclude that the results are within strictly defined tolerances, and the roller of this type is free of any surface and hidden defects. The calculations indicate that the service life of HSS rolls is 14,000–20,000 Mg of rolled material per 1 mm of wear on the roll surface in the direction of its radius, while up to 2000 Mg per 1 mm is obtained for high chromium cast iron rolls. The durability of the materials was also determined by microscopic examination of the working surface of the roll barrels. Cast iron rollers were compared with high-speed steel rollers [35,44]. The cross-section of the roll made of high chromium cast iron shown in Figure 9 illustrates that the cracks formed on the surface of the roll as a result of contact between the rolls and the heated metal band. On the other hand, the rolls made of high-speed steel, due to higher durability obtained because of the presence of harder carbides in their structure of the types MC, M_7_C and M_2_C, oppose the formation of this type of defect, which is illustrated in Figure 10. In addition, this calculation shows an 80% reduction in wear of HSS type rolls compared to high chromium rolls. A comparative analysis of the two tested roll grades is shown in Table 7. Rolls made of high chromium cast iron have such a low hardness on their working surface that they are unable to resist the natural operating conditions that occur during the rolling process. Therefore, they are easily susceptible to surface wear resulting from, among other things, the chemical composition of the rolled steel, temperature of the rolled material, values of unit and total pressures of applied metal on rolls, rolling speeds, values of slippage in the rolling basin of the rolled material, etc.

The implementation of HSS rollers in the examined hot rolling mill, apart from a pronounced increase in their durability, makes it possible to assume that it will also reduce the costs connected with the purchase of new rollers by about 30%, as after the roll has been used up, only the working sleeve has to be replaced and not the whole roll as is the case with rollers made of high chromium cast iron. The correct choice of chemical composition—in particular, the increase in the proportion of carbides and the correct structure of the material—has improved the durability of the roll, especially its hardness, wear resistance and the relevant surface roughness. For example, when rolling in the first frames of the finishing unit, a low surface friction is required, and in the last frames, a high resistance to sticking and cracking of the rolls. This means that the steel composition of the rollers must be differentiated for each cage. HSS rolls perform well, mainly in the last cages of the F5 and F6 finishing groups. Thanks to their properties, which are obtained by applying the appropriate production technology, among other things, the following has been achieved: minimum phosphorus, sulfur and gas content; a minimum of non-metallic inclusions; the finest possible grain size; the highest possible degree of uniformity of chemical composition and temperature. Thanks to the reduction of rolling mill stops caused by the necessity of replacing rolls in all cages, a reduction in the direct wear of rolls by abrasion and oxidation was achieved. Thus, the use of rolls made of high-speed steel, HSS, results in an increase in rolling efficiency and large economic benefits. With a proper operation, the strength of rolls made of HSS is several times higher than the strength of rolls made of high chromium cast iron, which influences the lengthening of time between necessary stoppages for roll replacement. In this way, not only is the cost of the rolls reduced, but the production preparation at the mill can also be planned more efficiently. For this reason, despite the significantly higher price of HSS rolls, it pays to employ them as their use results in measurable economic benefits.

## 5. Conclusions

Based on the analysis performed of the wear of rolls in the hot strip mill, the following conclusions can be made:

1. The statistical method for determining the service life of working rolls indicated that their reconstruction is determined both by natural physical phenomena and, in about 10% of cases, by their improper use, mainly in frames F5 and F6 of the finishing set.
2. Roll-wear indicators are of key importance for the construction of rolling campaigns and production schedules, decisively influencing the reduction of roll wear and costs and changeover times.
3. Calculations made using the program: “Roll Grinding Management System” indicate the possibility of replacing the working rolls of high chromium cast iron Hi-Cr with HSS rollers in cages F5 and F6, which will contribute to an increase in the hardness of the rolls, a reduction in production costs and a decrease in the number of rebuilds.
4. The operating life of HSS rolls is 14,000–20,000 Mg of rolled material per millimeter of wear on its surface in the radial direction, compared to 2000 Mg for rolls made of high chromium cast iron Hi-Cr.
5. HSS type rolls show 80% less wear compared to high chromium rolls. The improvement of the durability of the roll, especially its hardness, wear resistance and suitable surface roughness, was influenced by the appropriate choice of material and mainly by the increase in the proportion of carbides and the appropriate structure of the material.
6. The benefits of this work include: the determination of cost and energy relationships between individual profiles and steel grades in active production; the ability to track energy consumption and processing costs depending on the currently produced assortment; and the verification of market prices for individual grades and profiles of steel.

## Figures and Tables

**Table 1:** Chemical composition of the tested HSS ingot.

Elemental Content, %									
C	Mn	Si	P	S	Cr	Ni	Mo	W	V
1.15	0.96	0.87	0.05	0.02	2.6	1.3	4.2	4.1	3.9

Source: own. based on [38].

**Table 2:** Statistical distribution of the life of rolls.

Grade of Material	Cage Number	Average	Standard Deviation	N	$\chi_{\alpha }^{2}$	$\chi_{obl}^{2}$	Notes
S235 JR	RM	727.57	315.00	15	9.488	1.494	no reason to reject the null hypothesis
	F1	2285.19	1047.64	21	5.991	0.844	no reason to reject the null hypothesis
	F2	1628.36	526.00	30	7.815	6.138	no reason to reject the null hypothesis
	F3	1674.58	415.29	79	9.488	8.670	no reason to reject the null hypothesis
	F4	695.34	354.72	72	9.488	3.367	no reason to reject the null hypothesis
	F5	543.63	138.00	173	10.068	46.900	the null hypothesis should be rejected
	F6	526.50	109.00	169	11.070	15.841	the null hypothesis should be rejected

**Table 3:** Roll consumption rates for 2020 and 2021.

Roll Type		Material	Consumption for the Year	
			2020	2021
Pre-assembly cage rolls				
Thrust rollers	ø 1600 × 2500	Steel 3% Cr	0.06	0.040
	ø 1600 × 2500	Cast steel	0.353	0.361
∑ for resistance rollers			0.413	0.401
Work rollers	ø 1100	ICDP	0.105	0.157
	ø 1250 × 2500	EICDP	0.353	0.361
∑ for work rollers			0.458	0.518
Finishing unit cage rollers				
Cage F1–F3	ø 825 × 2500	ICDP	0.557	0.538
Cage F1–F3	ø 825 × 2500	ICDP	0.463	0.473
Cage F4	ø 825 × 2500	EICDP	0.152	0.191
Cage F4	ø 825 × 2500	ICDP	1.056	1.092
Cage F5 i F6	ø 730 × 2500	Hi-Cr	1.982	1.961
Cage F5 i F6	ø 730 × 2500	Hi-Cr	2.011	2.197
∑ for working and back-up rollers			6.221	6.452
∑ for work rolls and back-up rolls of the entire rolling assembly rolling mill unit			7.092	7.371

Source: own, based on [38].

**Table 4:** Results of hardness measurements of HSS specimen.

Type of Heat Treatment	Hardness		
	HB	HRC	HSh
Condition after casting	588–618	60	82–83
Softened 700 °C/8 h	267–278	-	55–58
Hardened 1050–1080 °C	-	61–63	77–80
Tempering once 580–600 °C/3 h	-	58–60	70–73
Double tempering 580–600 °C/3 h	-	50–52	71–75

Source: own, based on [38].

**Table 5:** Map of faults in the high chromium Hi-Cr cast roll, mm.

Roll Sectors	Roller Stations													
	1	2	3	4	5	6	7	8	9	10	11	12	13	14
	Roll Fracture Defect													
8	0.08	0.10	0.10	0.09	0.10	0.13	0.09	0.15	0.19	0.25	0.17	0.18	0.14	0.29
7	0.12	0.12	0.10	0.06	0.10	0.12	0.11	0.14	0.18	0.27	0.22	0.21	0.13	0.60
6	0.09	0.11	0.06	0.09	0.10	0.11	0.10	0.13	0.22	0.23	0.27	0.16	0.11	0.22
5	0.10	0.10	0.08	0.08	0.10	0.14	0.11	0.12	0.17	0.20	0.18	0.11	0.21	0.18
4	0.10	0.10	0.09	0.09	0.11	0.11	0.11	0.15	0.21	0.16	0.15	0.12	0.14	0.14
3	0.07	0.09	0.10	0.08	0.11	0.09	0.13	0.14	0.19	0.28	0.18	0.13	0.14	0.13
2	0.08	0.06	0.11	0.06	0.10	0.08	0.09	0.13	0.20	0.22	0.18	0.12	0.19	0.40
1	0.09	0.08	0.10	0.10	0.12	0.09	0.09	0.14	0.19	0.22	0.17	0.17	0.13	0.24
**Roll Sectors**	**Roller Stations**													
	**15**	**16**	**17**	**18**	**19**	**20**	**21**	**22**	**23**	**24**	**25**	**26**	**27**	**28**
	**Roll Fracture Defect**													
8	0.13	0.43	0.16	0.20	0.22	0.17	0.11	0.06	0.11	0.09	0.10	0.10	0.09	0.12
7	0.23	0.27	0.14	0.25	0.25	0.17	0.09	0.07	0.07	0.09	0.09	0.09	0.06	0.11
6	0.48	0.16	0.16	0.17	0.23	0.19	0.13	0.08	0.07	0.08	0.08	0.07	0.05	0.14
5	0.11	0.14	0.15	0.14	0.20	0.17	0.13	0.10	0.10	0.11	0.09	0.08	0.11	0.13
4	0.13	0.15	0.16	0.17	0.21	0.19	0.18	0.22	0.13	0.09	0.08	0.09	0.14	0.07
3	0.29	0.16	0.12	0.16	0.21	0.18	0.13	0.10	0.09	0.08	0.06	0.10	0.08	0.08
2	0.52	0.21	0.16	0.19	0.23	0.16	0.13	0.09	0.09	0.09	0.09	0.11	0.10	0.10
1	0.14	0.16	0.18	0.18	0.21	0.21	0.11	0.19	0.08	0.10	0.09	0.09	0.08	0.12

Source: own, based on [38].

**Table 6:** Map of defects for HSS type rolling.

Roll Sectors	Roller Stations													
	1	2	3	4	5	6	7	8	9	10	11	12	13	14
	Roll Fracture Defect													
8	0.19	0.18	0.13	0.18	0.20	0.11	0.19	0.12	0.15	0.19	0.11	0.08	0.17	0.16
7	0.18	0.17	0.14	0.18	0.52	0.19	0.18	0.09	0.13	0.17	0.17	0.11	0.15	0.11
6	0.16	0.11	0.18	0.17	0.21	0.18	0.19	0.11	0.16	0.15	0.19	0.16	0.11	0.15
5	0.11	0.13	0.17	0.18	0.24	0.17	0.18	0.16	0.17	0.12	0.21	0.17	0.18	0.17
4	0.12	0.17	0.15	0.15	0.11	0.15	0.15	0.17	0.11	0.18	0.19	0.15	0.14	0.13
3	0.10	0.15	0.09	0.11	0.17	0.19	0.16	0.17	0.10	0.16	0.17	0.11	0.16	0.11
2	0.12	0.15	0.05	0.16	0.23	0.11	0.14	0.19	0.08	0.14	0.14	0.13	0.14	0.15
1	0.13	0.18	0.11	0.13	0.22	0.12	0.16	0.19	0.11	0.11	0.10	0.09	0.12	0.14

Source: own, based on [44].

**Table 7:** Comparative analysis of the influence of the performance parameters of rollers made of grades Hi-Cr and HSS on the quality of a finished product.

Parameters	Roll Material	
	High Chrome Cast Iron Hi-Cr Type	High Speed Steel HSS Type
Hardness, HSh	56–64	78–81
Structure	martensitic containing 25–30% carbide M_7_C_3_	martensitic containing 40–55% carbide: MC, M_7_C, M_2_C
Application	the last cages of the rolling assembly	all rolling cages
Number of realized rolling campaigns in one roll development	1	7–8
Persistence, Mg	2000–3000	14,000–20,000
Mill yield, %	94.50	98.50
Roller wear, kg/Mg	2.20	0.64
Electricity consumption, kWh/Mg	92	71
Water consumption for cooling, m^3^/Mg	1.23	0.84

Source: own, based on [38].

## Data Availability

Not applicable.
